# Giant sacrococcygeal teratoma in an infant: a case report with a literature review

**DOI:** 10.1097/MS9.0000000000001274

**Published:** 2023-09-07

**Authors:** Wirya N. Sabir, Sasan M. Ahmed, Karzan M. Hasan, Bilal A. Mohammed, Honar Othman Kareem, Zana B. Najmadden, Berun A. Abdalla, Rawezh Q. Salih, Shvan H. Mohammed, Fahmi H. Kakamad, Hevar A. Azaldeen

**Affiliations:** a Smart Health Tower; b Kscien Organization for Scientific Research (Middle East office); c College of Medicine, University of Sulaimani, Sulaimani; d Research Center, University of Halabja, Halabja, Kurdistan, Iraq

**Keywords:** case report, coccygectomy, embryonal tumor, neonate, sacrococcygeal teratoma

## Abstract

**Introduction and importance:**

A sacrococcygeal teratoma (SCT) is a rare embryonal tumor that emerges in the sacrococcygeal area. It affects one in every 35 000–40 000 live births. Herein, we report a case of a substantial SCT in a neonate.

**Case presentation:**

A neonate girl from consanguineous parents was delivered by cesarean section with a large mass (18×17 cm) in the sacrococcygeal area. The baby’s birth weight was 5 kg, of which 2.5 belonged to the mass. The vital signs were within normal ranges and she had weak movement with bluish peripheral limbs. Oxygen saturation was around 85% for a short period after birth. According to the American Academy of Pediatric Surgical Section, the tumor was type I. After the fifth day of delivery, a complete resection was done through a chevron incision. The patient was put on ‘nil by mouth’ for about 24 h and given intravenous fluid.

**Clinical discussion:**

The histopathological examination of the surgical specimen confirmed extragonadal immature teratoma. The histological classification of SCT is divided into three types: malignant teratomas (consisting of malignant germ cells); immature teratomas (incompletely differentiated structures with a high risk of malignancy or embryonal components); and mature teratomas (fully differentiated tissues).

**Conclusion:**

SCT has rarely been reported as a giant mass. Radiologic examinations in the early stages of pregnancy may be essential to the early diagnosis of the condition.

## Introduction

Highlights

- Sacrococcygeal teratoma (SCT) is a rare form of embryonal tumor that emerges in the sacrococcygeal area.
- SCT affects about one in every 35,000–40,000 live births.
- This phenomenon is a very uncommon disease, and there are only a few reported cases in the literature.
- This study aims to report a rare case of a giant SCT in a neonate.

Sacrococcygeal teratoma (SCT) is a rare form of embryonal tumor that emerges in the sacrococcygeal area (SCA), containing tissues from all three germ layers (endoderm, mesoderm, ectoderm). SCT affects one in every 35,000–40,000 live births with a female-to-male ratio of 3:1 to 4:1^1^. Detecting first-trimester SCT relies on identifying a protruding mass in the caudal region. Some SCTs might be small during the first trimester, making them harder to detect early. Hence, only cases with large masses can be diagnosed in this period^2^. In addition, the routine sonographic examination in the second trimester aids in diagnosing the tumor to prevent perinatal and neonatal complications such as fetal hydrops, tumor rupture, high output cardiac failure, and placentomegaly, which are associated with increased mortality rate^3–5^. Clinical signs and symptoms such as anal displacement with the tightness of the anal canal and constipation can occur due to tumor compression on the bladder or rectum^4^. In 1973, based on the intrapelvic/intra-abdominal extension of the tumor and its external component, Altman classified SCT into four types, according to the American Academy of Pediatric Surgical Section (AAPSS)^6^. Herein, we report a case of giant SCT in a neonate. The report has been written according to the SCARE (Surgical CAse REport) guideline and the references have been confirmed to be eligible^7,8^.

## Case presentation

### Patient information

**Figure 1:**
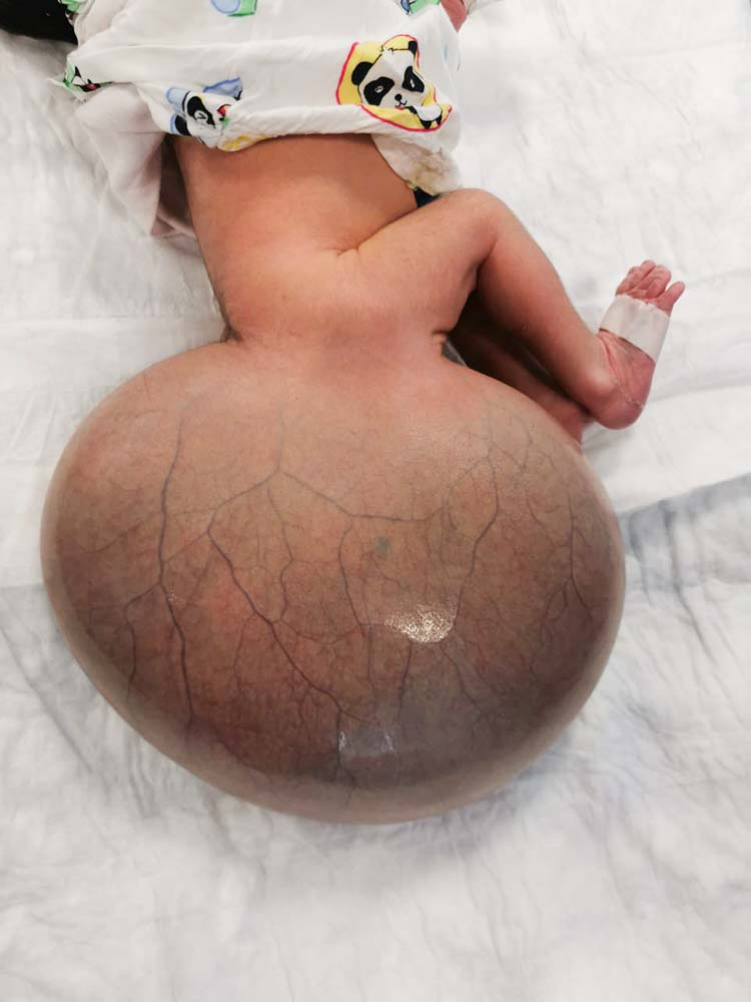
The neonate with giant sacrococcygeal teratoma in the sacrococcygeal area.

A neonate girl from consanguineous parents was delivered by cesarean section with a large mass (18×17 cm) in the SCA (Fig. 1). The mass was found after the first obstetric ultrasonography (U/S) in the second trimester because the parents omitted the first-trimester evaluation. We did not obtain a costly genetic profile, but the family history of the case was negative for genetic disorders. The mother of the case was overweight, with a body mass index of 30 kg/m^2^, without any chronic diseases.

### Clinical findings

During the cesarean section, no significant complications were encountered except for a brief period of blood pressure instability in the mother. The baby weighed 5 kg at birth. The patient’s heart rate (150 beats per minute) and respiratory rate (50 breaths per minute) were normal, while she had an oxygen saturation of around 85% for a short period after birth. She had weak movement and bluish peripheral limbs.

### Diagnostic assessment

**Figure 2:**
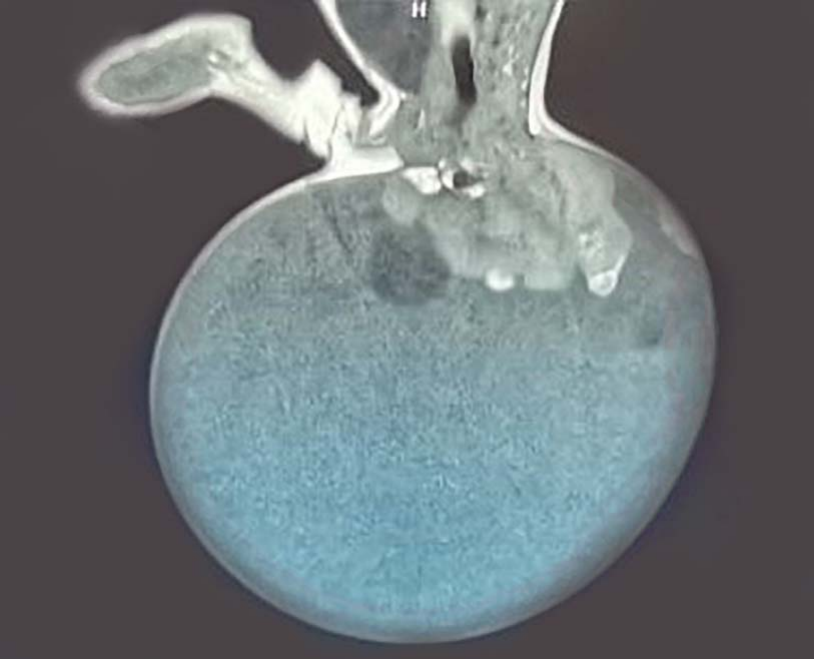
Magnetic resonance imaging showed a complete external component.

The mass weight was 2.5 kg, with a soft surface and variable consistency (Fig. 1). After delivery, Magnetic resonance imaging (MRI) was done for the infant. According to the AAPSS, the tumor was a type I since it had a complete external component (Fig. 2). Preoperatively, the blood investigations, including the complete blood count, were within normal ranges, and she was negative for hepatitis virus and elevated alpha-fetoprotein (AFP).

### Therapeutic intervention

The baby was admitted to the neonatal intensive care unit (NICU). An incubator was used to prevent hypothermia, oxygen therapy by nasal cannula, and for further monitoring and management.

**Figure 3:**
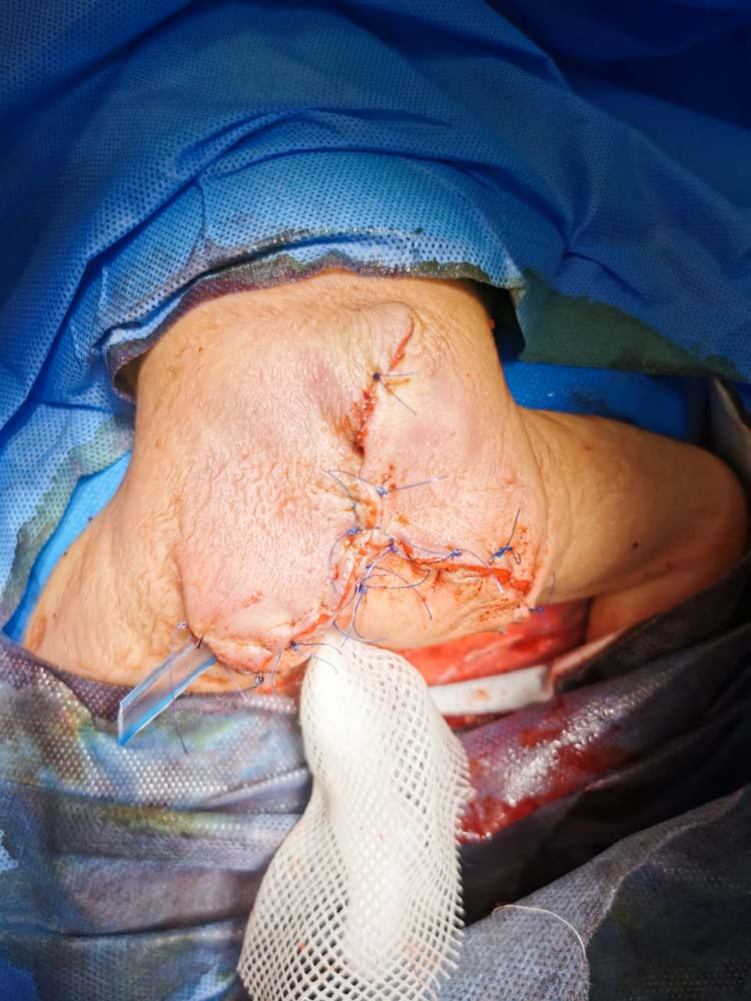
Postoperative outcome after complete resection of the mass through a chevron incision.

After the fifth day of delivery, under general anesthesia, a complete resection through a chevron incision was done. Intraoperatively, a Hegar dilator was placed inside the rectum as a guide, and a small lower lateral rectal tear was sutured without postoperative complication (Fig. 3). The patient received intravenous fluids and was put on nil by mouth (NBM) for ~24 h. On the second day after surgery, when the patient had a bowel movement, oral intake was initiated. The histopathological examination revealed an extragonadal immature teratoma.

### Follow-up

The postoperative period was uneventful, and the baby was discharged home after 7 days. After 2 years of follow-up, no recurrence was demonstrated.

## Discussion

The SCT is an infrequent embryonal tumor in the SCA of neonates. It has a favorable prognosis depending on the date of diagnosis, the simplicity of coccygectomy, and the tumor’s risk for malignancy. The female neonate is mainly affected; however, it is unclear why women predominate^2,9^. According to the AAPSS, there are four types of SCT based on internal and external tumors. Type I is characterized by an externally visible mass; type II by an external mass with a considerable intrapelvic component; type III by an external mass with a pelvic mass; and type IV by an entirely internal mass. Prenatal U/S makes identifying types I and II easy, while type IV has a higher risk of malignancy. The present case was a neonate female with a type 1 SCT that could easily be diagnosed by U/S examination during the second trimester. Regular U/S is often used in the second trimester to make an antenatal diagnosis, while detection is possible in the first trimester. It is common for pregnant women, and also in our case, to skip regular U/S screenings in the first trimester, which causes challenges in detecting masses at an early stage^3^. It has been reported that the detection of SCT in the first trimester may go unnoticed due to its small size^2^. However, in the present case, due to the large size, it would have been noticed in the first trimester. However, we believe that even if we had detected it in the first trimester, nothing significant would have changed in the therapeutic plan. Due to cultural beliefs, it was impossible for parents to accept termination during that period.

The Benachi classified the prognosis into three categories. Group A includes tumors smaller than 10 cm or avascular tumors moderately vascularized with gradual growth; group B includes tumors greater than 10 cm with rapid growth and marked vascularity linked to heart failure; and group C includes tumors of 10 cm or greater with gradual growth, cystic predominance, and moderately vascularization. The tumor of the present case was classified as group C^10,11^. The primary treatment for SCT is coccygectomy within a few days after delivery. The optimal time for surgery is between the infant’s birth and 2 months of age. The surgery time is essential since postponing is linked to a greater risk of malignancy and recurrence. Without coccyx excision, the recurrence rate reaches 37%^9,10^. Due to the above-mentioned risks, in the present case, a coccygectomy was performed on the fifth day after delivery.

Infants with large or severe vascular tumors should have a cesarean delivery to limit the risk of bleeding^3^. During the perinatal period, increasing tumor size may cause obstetric complications such as a premature baby, tumor hemorrhage or rupture, or dystocia^1^. In the present case, although the mother was overweight with some blood pressure instability, no complications occurred during and after birth. Body structural anomalies may be found due to the tumor’s growth, such as cardiomegaly, anal stenosis, and hydronephrosis. The size of the tumor and the pressure it puts on nearby organs may be detected by MRI, which can also differentiate this condition from common differential diagnoses such as distal neural tube defects (myelomeningocele or myelocystocele)^3^.

Recently, there has been an agreement that coccygectomy is the best way to avoid a benign teratoma recurrence with a decrease in any harm to the tumor cyst wall. However, even after complete excision, an SCT may return years later, so patients must be under continual monitoring till adulthood^3^. Although the literature has reported excellent survival rates, the mortality rate for masses larger than 10 cm is 18%. Even in patients with an entirely removed coccyx, the probability of recurrence ranges from 11% to 22%, so close follow-up every 3–6 months with physical examinations such as a rectal exam, diagnostic imaging, and AFP are recommended for at least 3 years^9^. In our case, there has been no recurrence after 2 years of follow-up.

High levels of tumor markers, such as AFP, are used to determine the probability of a malignant tumor. High levels of AFP may indicate malignancy. In newborn babies, these values are typically high and only return to normal levels around 9 months of age. Therefore, this may lead to misdiagnosing the condition as a malignant tumor. Due to this reason, an MRI was done for the present case to differentiate if the tumor was malignant or not^12^. The histological classification of SCT is divided into three types: malignant teratomas (consisting of malignant germ cells); immature teratomas (incompletely differentiated structures with a high risk of malignancy or embryonal components); and mature teratomas (fully differentiated tissues like hair, bone, and teeth). Histopathology results in our case confirmed an immature teratoma. The microscopy section in histopathology revealed mature tissues from all three germ layers. In the present case, AFP was elevated; however, MRI revealed that the mass was type I. According to the AAPSS. A complete resection of the mass was conducted through a chevron incision.

## Conclusion

SCT has rarely been reported as a giant mass. Radiologic examinations in the early stages of pregnancy may be essential to the early diagnosis of the condition. Despite the associated risks and complications, neonates with giant SCT can be delivered by cesarean section without significant complications.

## Ethical approval

The ethical committee of the University of Sulaimani does not need ethical approval for case reports (until 3 cases) in our locality.

## Consent

Written informed consent was obtained from the patient for the publication of this case report and accompanying images. A copy of the written consent is available for review by the Editor-in-Chief of this journal on request.

## Patient perspective

The patient was completely satisfied with the management approach and did not have any complaints about it. Additionally, the patient praised the staff for their efforts during his treatment.

## Sources of funding

None to be declared.

## Author contribution

W.N.S.: the pediatric surgeon who managed the case and was one of the major contributors to the study; K.M.H., B.A.M., and H.O.K.: conducted the literature review; S.M.A. and F.H.K.: involved in the manuscript drafting; B.A.A., S.H.M., Z.B.N., R.Q.S., and H.A.A.: involved in the critical review and improvement of the manuscript. All of the authors were involved in the final approval of the study.

## Conflicts of interest disclosure

The authors declare that they have no conflicts of interest.

## Research registration unique identifying number (UIN)

Our case does not match the criteria you have mentioned as it is a case report, so our study was not registered. If registration is still needed at the end, we will register the case at the following registry: http://www.researchregistry.com

## Guarantor

Fahmi H. Kakamad.

## Data availability statement

Not applicable.

## Provenance and peer review

Not commissioned, externally peer-reviewed.
